# Recent advances in eosinophilic esophagitis

**DOI:** 10.12688/f1000research.11798.1

**Published:** 2017-09-28

**Authors:** Sandy Durrani, Marc Rothenberg

**Affiliations:** 1 Division of Allergy and Immunology, Department of Pediatrics, Cincinnati Children’s Hospital Medical Center, University of Cincinnati, Cincinnati OH, 45529, USA

**Keywords:** Eosinophilic esophagitis, Proton-Pump inhibitor, Topical Croticosteroid

## Abstract

Eosinophilic esophagitis is a chronic, antigen-driven, eosinophil-predominant inflammatory disease of the esophagus and affects both children and adults. Cutting-edge technologies, such as genome-wide association studies, have advanced our understanding of the disease pathogenesis at a remarkable rate. Recent insights from genetic and mechanistic studies have concluded that a complex interplay between genetic and environmental risk factors, allergic sensitization, and esophageal-specific pathways leads to disease pathogenesis. Importantly, recent epidemiologic studies have found that the incidence and prevalence of eosinophilic esophagitis continue to rise. New guidelines have advocated the elimination of the term proton pump inhibitor (PPI)–responsive esophageal eosinophilia and have recommended using PPIs as a first-line treatment modality. Systemic reviews and meta-analyses confirm the efficacy of PPIs, topical corticosteroids, and empiric food elimination diets. Unmet needs include the development of birth cohort studies, validated diagnostic scoring systems, minimally invasive disease-monitoring methods, and the development of new therapies.

## Introduction

Eosinophilic esophagitis (EoE) is a fascinating case study in modern medicine today as it was only recently recognized in the 1990s as a clinicopathologic disorder—a metaphorical infant when compared with other allergic diseases such as asthma and allergic rhinitis. Importantly, owing to the development and use of cutting-edge molecular diagnostic technology, the scientific breakthroughs in our understanding of EoE rival those of diseases recognized centuries ago. Nonetheless, much work remains to be done to understand this enigmatic food antigen–induced disorder of chronic esophageal inflammation of both children and adults. If left untreated, EoE can progress over time to a fibrostenotic disorder characterized by dysphagia and esophageal strictures. As a result of the overwhelming number of genetic, mechanistic, and clinical breakthroughs in EoE over the last two decades, the research and medical communities are in need of frequent and comprehensive reviews of the literature by international experts. Thus, the following review highlights and summarizes important epidemiologic, genetic, pathophysiologic, and clinical findings over the last three years.

## Epidemiology

The incidence and prevalence of EoE have been rising sharply over the last 20 years in Western countries ^1^. Indeed, numerous studies seem to confirm a rapid rise in the incidence and prevalence of EoE ^2^. Recently, a systematic review of 13 population-based studies confirmed a higher incidence (7.2 per 100,000 person-years) and pooled prevalence (28.1 cases per 100,000 inhabitant-years) comparing studies before and after 2008. Moreover, the incidence and prevalence of EoE were higher in adults than in children and the incidence of EoE was higher in the US than in Europe ^3^. Finally, there are also now reports of EoE cases in Africa—a heretofore undocumented finding ^4^.

EoE has been classically observed in studies to be a disease of Caucasian males ^2^; however, two recent studies have found that African-American male children have the expected burden of EoE seen in Caucasians ^5, 6^. Weiler *et al*. ^6^ found that African-American children presented at younger ages with higher rates of failure to thrive, vomiting, and atopic dermatitis. Moreover, an older retrospective study of 208 EoE cases in North Carolina (US) found no differences in clinical, endoscopic, and histologic features by either race or gender ^7^. Furthermore, African-Americans were more likely to present earlier with failure to thrive whereas Caucasians were more likely to present with dysphagia and esophageal rings ^7^. Taken together, these studies suggest that African-Americans may develop EoE more frequently and more aggressively than previously reported ^5– 7^. However, a recent population-based study of an electronic medical database in the US demonstrated that Caucasians are affected at higher rates than African-Americans ^8^. Regardless, an important area of future research is to confirm racial EoE phenotypes and determine whether racially and ethnically unique mechanisms underpin disease presentation and treatment response and influence health-care disparities similar to asthma.

EoE has been found to predominantly affect males in numerous studies with estimates as high as 70–85% ^2, 9^. A recent meta-analysis found that the risk for EoE was significantly higher for males ^3^. Moreover, a retrospective, multicenter, cross-sectional analysis of 793 patients with EoE found that the disease predominantly affected male patients (72%) ^10^. There were no differences in clinical, endoscopic, and histologic characteristics except that a higher percentage of males had strictures ^10^. Importantly, a smaller recent retrospective case control study found that adult women with EoE were more likely to report chest pain (as well as a non-significant trend toward more heartburn symptoms) but that adult males with EoE were more likely to report dysphagia and food impactions ^9^. It is possible that women may be underdiagnosed due to displaying more inflammatory symptoms such as chest pain and heartburn rather than the classic symptoms of dysphagia and food impactions of EoE. Indeed, males may have more fibrostenotic symptoms and thus are more easily diagnosed. Finally, the authors astutely point out that their study is one of the few focused on gender differences in EoE suggesting gender health-care disparities ^9^.

## Pathophysiology

Elucidating the heritability and pathophysiology of EoE via genetic risk variants and molecular pathways has involved the use of cutting-edge technologies such as genome-wide association studies (GWASs), candidate gene studies, epigenomics, and DNA methylation profiling and chromatin immunoprecipitation sequencing technologies ^11^. The factors that contribute to the risk of EoE are genetics as well as early-life environmental exposures (for example, antibiotic use in infancy, caesarean delivery, preterm birth, season of birth, breastfeeding, and exposures affecting the microbiome) ^12– 14^. Disease inception involves a complex interplay between epithelial inflammatory pathways, impaired barrier function, and dysregulated transforming growth factor beta (TGF-β) activity/production and activity and induction of allergic TH _2_ inflammation mediated by eosinophils and mast cells ^12^. Although a detailed discussion on disease pathophysiology is beyond the scope of this review, we will highlight two recent discoveries involving thymic stromal lymphopoietin (TSLP) and calpain 14 (CAPN14).

An important reason why our understanding of the EoE disease inception and pathophysiology has progressed rapidly is the use of GWASs. These studies allow disease risk variants to be identified in a more unbiased fashion. A previous GWAS has identified a strong association between EoE and the 5q22 locus, which spans the TSLP domain ^15^. Importantly, TSLP is a potent mediator of TH _2_ allergic responses and basophil activation ^16^. Furthermore, a separate candidate gene approach found a coding variant in the gene encoding the receptor for TSLP (cytokine receptor-like factor 2, or CRLF2), which is (fascinatingly) associated with EoE risk in men only ^17^. This represents a possible explanation for why risk of EoE is significantly higher in men (approximately 3:1 male:female) ^3^.

**Figure 1.**
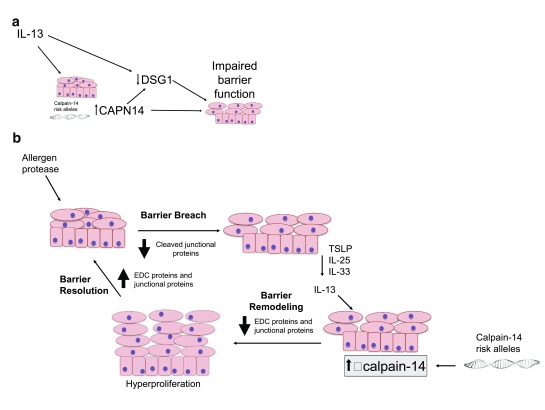
Interleukin-13 (IL-13) induces calpain 14 (CAPN14) effector and regulatory roles in genetically predisposed patients. An important function in eosinophilic esophagitis (EoE) disease pathogenesis involves IL-13 stimulating CAPN14 expression and desmoglein 1 (DSG1) downregulation ( **a**). CAPN14 and IL-13 reduce DSG1 expression, which leads to decreased barrier function and likely increased TH _2_ responses seen in EoE ( **b**). Adapted from Davis *et al*. ^20^. EDC, epidermal differentiation complex; TSLP, thymic stromal lymphopoietin.

An independently confirmed ^18^, more recent GWAS was performed on 2.5 million genetic variants of 726 EoE cases and 9,246 controls. In addition to reproducing EoE risk association at the 5q22/ *TSLP* locus, the authors found a strong association at 2p23, which encodes CAPN14 ^19^. CAPN14 is an esophagus-specific intracellular epithelial protease that is induced by interleukin-13 (IL-13) (and genetic variants are risk factors for EoE). Although its role is still being defined, CAPN14 is thought to directly and indirectly (through desmoglein 1) impair epithelial barrier function in EoE ( Figure 1) ^20^. Importantly, *CAPN14* is expressed exclusively in the esophagus when compared with 130 other tissues—a potentially remarkable breakthrough. The *CAPN14* risk variant represents evidence of esophageal-specific pathways in EoE disease pathophysiology and has potential in future, targeted therapeutic applications in EoE. As a result of the above findings, a two-hit mechanism of EoE susceptibility has been proposed ^12^. The first hit occurs at 5q22, leading to TSLP-induced allergic sensitization. The second hit occurs at 2p23, leading to activation of CAPN14—a potent esophageal-specific protease now shown to regulate epithelial cell barrier function ^12, 19, 20^.

## Diagnosis

The diagnostic criteria for EoE have evolved but mainly required symptoms of esophageal dysfunction with histologic evidence of eosinophil-predominant inflammation consisting of a peak value of at least 15 eosinophils per high-power field. Prior to 2011, the mucosal eosinophilia had to be isolated to the esophagus and persist after 8 weeks of treatment with a proton pump inhibitor (PPI) trial to meet diagnostic criteria ^21, 22^. Since 2011, PPI-responsive esophageal eosinophilia (PPI-REE) has been recognized as a specific disease phenotype in which patients respond clinically and histologically to PPI ^21^. However, there is strong evidence that PPI-REE and EoE share overlapping genetic, molecular, histologic, and endoscopic features to suggest that the two are of the same disease spectrum ^2, 23^. Therefore, the recently published European guidelines recommended retraction of the term PPI-REE and further recommended that PPI be considered a first-line treatment for EoE rather than a diagnostic criterion ^2^.

An important unmet need has been the development of validated scoring systems for additional histologic features besides peak eosinophil counts. Recently, Collins *et al*. ^24^ developed and validated an EoE histologic scoring system (EoEHSS) taking into account eight EoE-associated features, including eosinophil density and basal zone hyperplasia. Interestingly, the EoEHSS better discriminated treated versus untreated patients compared with peak eosinophil counts ^24^. Importantly, this study underscores the observed discordance between histology and symptoms clinically and meets an important need to develop prospectively validated instruments to better characterize whether symptoms associate with histology/disease activity. Indeed, it has been difficult to evaluate symptom-histology associations without a standardized scoring system to compare and confirm findings from separate studies ^25^. Interestingly, a recent multicenter prospective study found only modest predictive capacity of the EoE Activity Index in predicting remission ^26^.

Finally, and perhaps most importantly, there is still a glaring lack of minimally invasive methods to monitor EoE disease activity. This unmet need remains of paramount importance as patients continue to be subjected to repeat endoscopies in order to diagnose, monitor, and effectively treat EoE. Moreover, repeat endoscopies significantly impact quality of life and health-care costs. Unfortunately, potential biomarkers of interest have not correlated with endoscopic and histologic findings thus far ^2^, although a transcriptome analysis of esophageal biopsies that has shown a sensitivity of 96% and a specificity of 98% for EoE is promising. Furthermore, the transcriptome analysis performs well with only one biopsy but still requires endoscopy to obtain tissue. There is emerging evidence that assessing mucosal integrity using endoscopic impedance measurements correlates with esophageal eosinophilia and may have value in disease management ^27, 28^. Finally, the String Test ^29^ and the CytoSponge ^30^, in which surface esophageal samples are retrieved with these semi-invasive devices, have shown promise in smaller studies. Perhaps using either the String Test or the CytoSponge in combination with the transcriptome analysis is the future of EoE disease surveillance and management.

## Treatment

**Figure 2.**
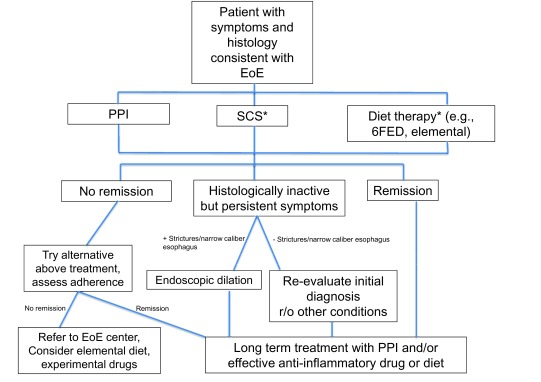
Treatment algorithm for patients with confirmed eosinophilic esophagitis (EoE). Adapted from European consensus guidelines ^2^. *Proton pump inhibitor (PPI) is first-line but consider swallowed corticosteroids (SCS) if there are severe symptoms, failure to thrive, or provider-patient preferences. *Can be done in addition to PPI or other drug or diet therapies. FED, food elimination diet.

The goal of therapy in EoE is to reduce inflammation and halt long-term progression to a fibrostenotic state. The established first-line treatments for EoE are PPIs, topical corticosteroids (CSs), and empiric food elimination diets. PPIs have recently been confirmed to establish histologic remission and symptom improvement in 50% and 60% of patients with EoE, respectively ^2, 31^. However, this meta-analysis should be interpreted with caution, as many of the studies were of poor quality ^2^. Nonetheless, the new, recently published European guidelines developed a treatment algorithm for EoE with PPIs considered as a first-line treatment for EoE; an evaluation and treatment algorithm is summarized in Figure 2 ^2^. Interestingly, PPIs block signal transducer and activator of transcription 6 (STAT6) activation and IL-13–induced signaling, which reduces eotaxin-3 production and consequently may have an anti-eosinophil effect ^32^. This mechanism, along with improvement of barrier function, may be the reason why PPIs are an effective form of treatment for some EoE cases ^12, 32^. A new and potent potassium channel acid blocker called vonoprazan has also shown evidence of clinical and histologic responsiveness in EoE ^33^.

The numbers of individual patients in each EoE trial are quite low; however, the cumulative numbers across all studies are now more substantial and have allowed insightful systematic reviews and meta-analyses to be performed ^2^. Multiple systematic reviews and meta-analyses have confirmed the efficacy of topical CSs for histologic remission EoE ^34– 37^; however, two of these reviews of the literature ^36, 37^ did not find evidence of symptom improvement with topical CSs. This disconnect may be due in large part to the use of different, non-validated, symptom-scoring tools for each study as well as overall small sample sizes ^2^. A recent prospective study in children found that 63% of children had sustained remission at 2 years of treatment with swallowed CSs, suggesting along with other studies that topical CSs can achieve long-term remission ^2, 38^. Finally, although swallowed budesonide and fluticasone are almost always prescribed, no formulation of topical CSs has been approved for EoE. Two different budesonide formulations—an effervescent tablet for orodispersible use (BET) and a viscous suspension (BVS)—are currently in clinical trials ( https://clinicaltrials.gov/ct2/show/NCT02493335?recrs=a&cond=Eosinophilic+Esophagitis&draw=1&rank=9 and https://clinicaltrials.gov/ct2/show/NCT02736409?recrs=a&cond=Eosinophilic+Esophagitis&draw=2&rank=11). Both BET and BVS have been found to be highly safe and effective thus far ^39^.

In terms of safety, a recent meta-analysis observed that topical CSs were not associated with significant adverse events other than a risk for developing asymptomatic esophageal candidiasis ^2, 37^. However, a study out of Cincinnati found that 10% of children whose EoE was treated with fluticasone (>440 μg daily) developed evidence of adrenal suppression ^40^. Moreover, another recent study found decreased cortisol stimulation with no evidence of adrenal insufficiency or growth delay in children on oral viscous budesonide ^41^. Many patients with EoE are on other forms of steroids for comorbid allergic conditions; therefore, more studies are needed to characterize the risk of adrenal suppression with topical CSs.

Recently, a meta-analysis found a 72% histologic remission rate using an empiric six-food elimination diet (6FED) ^3^. Importantly, there are trials ongoing to understand whether less restrictive elimination diets can be used. One-food (1FED; milk), two-food (2FED; milk and wheat), and four-food (4FED; milk, wheat, soy, and eggs) elimination diets are currently being investigated ( https://clinicaltrials.gov/ct2/show/NCT02610816) ^2^. Specifically, a multicenter trial of a 4FED (milk, wheat, egg, and soy) was found to induce remission in 60% of children with EoE ^42^. Furthermore, a recent study found that 61% of children had histologic remission with elimination of milk only ^43^. Initial empiric elimination diets that are less restrictive have the potential advantages of reducing the number of endoscopies while improving nutrition and quality of life.

Finally, the cumulative evidence is leading many to conclude that food allergy testing-directed elimination diets have little role in the management of EoE ^2, 44^. Indeed, at Cincinnati Children’s Hospital Medical Center, clinicians who routinely care for patients with EoE do not test for causative food triggers by using skin tests, *in vitro* IgE measures, or patch testing unless there is clear evidence of an IgE-mediated reaction (for example, hives and anaphylaxis). It is the authors’ opinion that the totality of evidence is fairly conclusive that test-directed elimination approaches are inadequate to identify food triggers in EoE, lead to confusion, and consequently delay attaining histologic and clinical control and remission.

## Conclusions

Our understanding of EoE is exponentially increasing. This is primarily due to cutting-edge technologies that have allowed researchers to discover disease mechanisms at an astonishing rate. A multi-hit mechanism of EoE pathogenesis involving a complex interplay between genetic and early-life environmental risk factors, allergic sensitization, and esophageal-specific pathways has been proposed ^12^. These pathways will offer new insights into mechanisms and potentially new therapeutic applications. The use of transcriptome analyses may further allow clinicians to “personalize” treatment depending on endotype ^12^. Unmet needs include developing and following high-risk birth or early-life cohorts prospectively over decades to understand disease inception, natural history, and sequelae. Finally, comprehensive standardized scoring systems for symptoms, quality of life, endoscopy, and histology need to be prospectively and universally validated. The recent formation of the Consortium of Eosinophilic Gastrointestinal Disease Researchers, which is part of the Rare Disease Clinical Research Network of the National Institutes of Health, provides an opportunity to unite clinicians, scientists, and a full spectrum of key stakeholders, including patients, to better understand and treat EoE ( https://www.rarediseasesnetwork.org/cms/cegir/).
